# The Promise of Adjunct Medications in Improving Type 1 Diabetes Outcomes: Glucagon-Like Peptide Receptor Agonists

**DOI:** 10.1177/19322968241309896

**Published:** 2024-12-31

**Authors:** Sujatha Seetharaman, Eda Cengiz

**Affiliations:** 1 Division of Pediatric Endocrinology & Diabetes, Department of Pediatrics, University of California San Francisco, San Francisco, CA, USA

**Keywords:** type 1 diabetes, liraglutide, semaglutide, tirzepatide, glucagon-like peptide receptor agonists

## Abstract

Type 1 diabetes (T1D) necessitates lifelong insulin therapy due to the autoimmune destruction of insulin-producing pancreatic beta cells. Despite advancements in diabetes technology and insulin formulations, maintaining optimal glycemic outcomes remains challenging in these individuals. Obesity, accompanied by insulin resistance, is common not only in type 2 diabetes (T2D) but also in many individuals with T1D. Glucagon-like peptide-1 receptor agonists (GLP-1 RAs), approved for T2D and obesity, are now being explored for off-label use in individuals with T1D. This review examines their efficacy, safety, and potential benefits in T1D management. We reviewed articles published up to May 2024 from databases like PubMed and Scopus, mainly focusing on human studies of GLP-1 RAs in T1D, as well as cardiorenal and metabolic outcomes in individuals with T2D and obesity. Semaglutide and other GLP-1 RAs showed significant improvements in glycemic outcomes, hemoglobin A_1c_ levels, reduced insulin doses, and notable weight loss. Studies in individuals with obesity and T2D showed significant improvements in lipid profile and offered cardiorenal protection. Common side effects include gastrointestinal issues, and while some studies reported hypoglycemia, hyperglycemia, and ketosis, others did not. Despite these challenges, GLP-1 RAs offer significant therapeutic benefits, making them a promising adjunct to insulin therapy for improving clinical outcomes in T1D management.

## Introduction

Type 1 diabetes (T1D) is an autoimmune disease marked by the destruction of insulin-producing pancreatic beta cells, necessitating lifelong dependence on exogenous insulin. This condition presents substantial public health and clinical challenges, with diagnosis rates increasing annually.^1-5^ Effective management requires precise insulin therapy to prevent ketoacidosis and maintain normal metabolic function, alongside frequent blood glucose monitoring to optimize glycemia and prevent complications.

Since the discovery of insulin over a century ago, significant strides in treatment have transformed care for countless individuals living with T1D. Despite advancements in insulin formulations, delivery systems, and continuous glucose monitoring (CGM), maintaining optimal glycemia remains challenging.^6^ Data from the T1D registry indicated that only a minority of individuals with T1D achieve the American Diabetes Association (ADA) hemoglobin A_1c_ (HbA1c) goal, with particularly high HbA1c levels observed in adolescents and youth.^7,8^ Furthermore, recent studies highlight significant long-term complications including brain changes^9^ and reductions in life expectancy for individuals with T1D, underscoring the urgent need for improved management strategies.^10,11^

The T1D is a significant burden for children and families, being one of the most common chronic childhood diseases.^12^ Despite advancements, there has not been a significant breakthrough in treating T1D. It is critical to develop interventions that can halt or slow disease progression and preserve residual beta-cell function while addressing abnormal physiology and factors causing beta-cell stress. Recently, new tools, such as glucagon-like peptide-1 receptor agonists (GLP-1 RAs), effective nutrient-stimulated hormone-based antiobesity pharmacotherapeutics, have emerged and are Food and Drug Administration (FDA) approved for the treatment of type 2 diabetes (T2D) and obesity.^13^

Recent research has explored the use of GLP-1 RAs as an adjunctive pharmacotherapy for individuals with T1D.^14,15^ Obesity, accompanied by insulin resistance, is prevalent not only in T2D but also in many individuals with T1D.^16-20^ Although GLP-1 RAs are not FDA-approved for T1D, several providers have been prescribing these medications off-label, especially for adults. This narrative review examines the emerging role of GLP-1 RAs in the management of T1D among both adults and adolescents, focusing on their efficacy, safety, and potential benefits.

## Methods

We reviewed articles published up to May 2024 using databases such as PubMed, Scopus, and ClinicalTrials.gov, with keywords including “Type 1 Diabetes,” “GLP-1 receptor agonists,” “liraglutide,” “Semaglutide,” “Tirzepatide,” “Exenatide,” and “Dulaglutide.” Pertinent articles’ references were also manually searched for relevant papers. We included papers involving human subjects and focused on the use of GLP-1RAs in T1D. Excluded were animal studies and non–peer-reviewed articles.

## The Potential of Glucagon-like Peptide-1 Receptor Agonists in Adolescents With Type 1 Diabetes

The GLP-1 RAs exhibit several mechanisms of action beneficial for managing diabetes. These include enhancing insulin secretion in response to hyperglycemia, suppressing glucagon secretion during hyperglycemia and euglycemia, slowing gastric emptying to stabilize post-meal glucose levels, and promoting weight loss through direct action on the brain^13,21^. Currently FDA-approved GLP-1 RAs among adults and adolescents are summarized in Table 1.

**Table 1.** Currently Approved GLP-1 RAs in Adults and Adolescents.

Medication	Year of FDA approval	US FDA indication	Mechanism of action	Side effects	Contraindications
Tirzepatide Initiated at 2.5 mg weekly; titrated to target dose, available as 5, 7.5, 10, and 12.5 with max dose 15 mg weekly, subcutaneously	Adults: Mounjaro for T2D (2022) Zepbound (2023) for obesity	T2D and/ or obesity in adults	Glucose- dependent insulin secretion slows gastric emptying, suppresses glucagon, and directly acts on the brain to decrease appetite	GI side effects (nausea, vomiting, diarrhea, constipation, decreased appetite), hypoglycemia when used with insulin, renal impairment or kidney failure, hypersensitivity, acute gallbladder disease	Personal history of pancreatitis History of Multiple Endocrine Neoplasia (MEN) type 2 Family history of medullary thyroid cancer Pregnancy
Semaglutide 0.25, 0.5, 1, 1.7, and 2.4 mg weekly (Wegovy), subcutaneously	Adults: Ozempco, 2017 Wegovy, 2021 Adolescents: Wegovy, 2022	Adult obesity, T2D Obesity^a^ in adolescents ≥12 years			
Oral semaglutide 3, 7, and 14 mg daily (Rybelsus)	Adults: Rybelsus, 2019	T2D in adults			
Liraglutide (Victoza 0.6, 1.2, and 1.8 daily)^22,23^ (Saxenda 0.6, 1.2, 1.8, 2.4, and 3 mg daily) subcutaneously	Adults: Victoza, 2010 Saxenda,2014 Adolescents: 2019 (Victoza) 2020 (Saxenda)	T2D in children ≥10 years Obesity in adolescents ≥12 years			
Dulaglutide 0.75, 1.5, 3, and 4.5 mg weekly, subcutaneously	Adults: Trulicity, 2014 Adolescents: Trulicity, 2022	T2D in adults T2D in children ≥10			
Exenatide, Byetta (short-acting formulation) 5, 10 mcg twice daily, or Bydureon (extended release) 2 mg weekly, subcutaneously	Adults: Byetta, 2005 twice daily Adolescents: Bydureon once weekly 2021	T2D in adults and children ≥10 years			

a BMI at or above the 95th percentile for their age and sex.

Given their effectiveness in reducing HbA1c and promoting weight loss without the risk of hypoglycemia, GLP-1 RAs are recommended as adjunct medications for adults and youth with T2D.^24^ However, there are currently no established guidelines for using GLP-1RAs in T1D due to the lack of robust studies, with limited research primarily focused on adults.

## Rationale for Use of Glucagon-like Peptide-1 Receptor Agonists as Adjunct Therapies in Adults and Adolescents With Type 1 Diabetes

### Glucagon-like Peptide-1 Receptor Agonists as a Tool to Manage Glycemic and Weight Outcomes in Type 1 Diabetes

Research indicates that T1D is characterized not only by insufficient insulin but also by an inappropriate glucagon response, contributing to postprandial hyperglycemia (Table 2).^38^ This dysregulation is commonly attributed to the absence of insulin secretion, which normally suppresses glucagon release.^38^ Studies show that individuals with T1D can suppress glucagon secretion after intravenous glucose administration, suggesting that inappropriate glucagon responses are likely triggered by oral glucose intake through gut signaling or direct gut glucagon secretion rather than alpha cell dysfunction or lack of insulin’s paracrine inhibition.^22,39,40^

**Table 2.** Overview of Trial Characteristics and Main Findings.

Study	Condition	Duration	N	Mean age	Duration of diabetes	BMI	Results
A. Studies evaluating glycemic control							
Liraglutide							
Mathieu et al^25^	T1D	52 weeks, mean HbA1c 8.2% Participants randomized to receive once-daily injections of liraglutide (1.8, 1.2, or 0.6 mg) or placebo added to insulin	1389	43.7 years, 47.7% male	24.1 years	29.5 kg/m^2^	Liraglutide added to insulin therapy reduced HbA1c levels (−0.34% to 0.54%), total insulin dose, and body weight. Rate of symptomatic hypoglycemia increased in all liraglutide groups
Ahren et al^26^	T1D	Liraglutide, 26 weeks RCT Mean HbA1c 8.1%	831	43.2 years, 46% male	21.1 years	28.9 kg/m^2^	Liraglutide reduced HbA1c, body weight, daily insulin use, increased rates of symptomatic hypoglycemia and hyperglycemia with ketosis compared to placebo
Kuhadia et al^27^	T1D	12-week RCT; participants on Liraglutide plus insulin randomized to receive either dapagliflozin 10 mg or placebo. Mean HbA1c 7.6%	30	44.8 years, 44.4% male	24.1 years	28.8 kg/m^2^	Addition of dapagliflozin to insulin and liraglutide group resulted in a reduction in HbA1c, caused weight loss, while increasing ketosis
Exenatide							
Herold et al^28^	T1D	24-week RCT Mean HbA1c 7.6%; participants randomized to receive exenatide 2 mg weekly vs placebo	79	36.1 years, 31.6% male	19.6 years	29.4 kg/m^2^	HbA1c levels were significantly reduced in the exenatide group after 12 weeks but no difference between the groups at 6 months. Weight loss seen in exenatide group, but no difference in hypoglycemia between the groups
Semaglutide							
Garg et al^29^	T1D	Retrospective study; participants on semaglutide for at least 3 months were followed for one year and compared with a control group; mean HbA1c 7.6 ± 1.2 in semaglutide group	50	42 ± 11 years, 30% male	27 ± 12 years	33.5 ± 5.8 kg/m^2^	At 1 year, semaglutide group had significant weight loss, improved glycemic metrics (HbA1c, CGM TIR, and glycemic variability), no change in insulin dose between the two groups
Case report: Raven et al^3^	T1D	Semaglutide given at a dose of 0.25 mg weekly and increased to 0.5 mg weekly	1	36-year-old female	27 years	29.3 kg/m^2^	At six months, patient had weight loss of 16 kg, insulin dose decreased by 36%, HbA1c improved, glycemic variability reduced, no increase in hypoglycemia
Letter to the Editor NEJM: Dandona et al^30^	T1D	Retrospective analysis: semaglutide started within three months after T1D diagnosis, followed for one year. Mean HbA1c 11.7% ± 2.1% at diagnosis	10		Between 21 and 39 years		Prandial insulin was eliminated in all the patients within three months, and basal insulin was eliminated in seven patients within six months. These doses were maintained until the end of the 12-month follow-up period. The mean HbA1c reduced to 5.9% ± 0.3% at six months and to 5.7% ± 0.4% at 12 months. Mild hypoglycemia occurred during the semaglutide dose increase. After stabilization, no DKA, hypoglycemic episodes, or other serious side effects
Meta-analysis							
Park et al^31^	T1D	24 studies using four different GLP-1 analogs (liraglutide, exenatide, albiglutide, lixisenatide), for 12 weeks	3377	39.3 years, 54.8% male	15.8 years	26.4 kg/m^2^	Liraglutide reduced A_1c_ (−0.09%/mg), weight (−2.2 kg/mg), and TDI (−4.32 IU/mg), with higher doses causing more nausea (OR 6.5), and ketosis (OR 1.8), but no significant increase in hypoglycemia risk
Tan et al^32^	T1D	11 RCTs included Mean HbA1c 8.1% (SD 1.0%)	2856	43.4 (SD 13.6) years, 52% female	22.6 years	29.2 (SD 5.3) kg/m^2^	Among individuals with obesity, GLP-1 RA and insulin combination resulted in improvement of metabolic profile, HbA1c reduction (−0.43%), weight loss (−6.28 kg), a lower insulin dose, and lower blood pressure. No increase in the incidence of severe hypoglycemia, diabetic ketoacidosis, or severe adverse events
B. Studies evaluating weight and cardiovascular outcomes							
Semaglutide							
Weghuber et al^33^	Obesity, adolescents	68-week RCT. Participants randomized to receive Semaglutide 2.4 mg weekly vs placebo	201	15.4 ± 1.6 years, 62% female	NA	37.0 ± 6.4	Mean change in BMI at week 68 was −16.1% with semaglutide and 0.6% with placebo. 73% participants in semaglutide group had weight loss of 5% or more compared to 18% in placebo group
Wilding et al^34^	Obesity, adults	68-week RCT; participants randomized to receive Semaglutide 2.4 mg weekly vs placebo	1961	46 years, 73.1% female	NA	37 kg/m^2^	Mean change in body weight from baseline to week 68 was −14.9% in the semaglutide group as compared with −2.4% with placebo
Lincoff et al^35^	Obesity, pre-existing CVD in adults, no diabetes	104-week RCT; participants randomized to receive Semaglutide 2.4 mg weekly vs placebo	17 604	61.6 ± 8.9 years, 72.3% male	NA	33.3±5.0	Semaglutide was superior to placebo in reducing the incidence of death from cardiovascular causes, nonfatal myocardial infarction, or nonfatal stroke
Navodnik et al^4^	T1D, no CVD	12-week RCT; Participants randomized to receive empagliflozin or semaglutide with a control group	89	48 years	21 years	28 kg/m^2^	Improvement in FMD (brachial artery flow-mediated dilation) was significant in both intervention groups compared to controls. Arterial stiffness improvements were seen only in the semaglutide group, with a decline in peripheral resistance by 5.1%
Tirzepatide (TZT)							
Jastreboff et al^36^	Obesity, adults	72-week RCT, phase 3, participants assigned in a 1:1:1:1:1 ratio to receive TZT, 5, 10, 15 mg or placebo for 72 weeks, including a 20-week dose escalation period	2539	44.9 years, 67.5% female	NA	38 kg/m^2^	Mean percentage change in weight was −15% with 5 mg, −19.5% with 10 mg, and −20.9% with 15 mg dose of TZT and −3.1% with placebo
Taktaz et al^5^	Adults with or without diabetes. Enrolled participants from the SURPASS-4 study, the SURPASS Clinical Trials Program SURMOUNT-1 trials	Meta-analysis, assigned to TZT vs placebo. Investigated effects of TZT on human cardiac AC16 cell lines, that were exposed to TZT under normal and high glucose conditions for seven days	7778	NA	NA	NA	Reduction in MACE compared to placebo (hazard ratio was 0.59). In AC16 cardiac cells, T reduced fibrosis, hypertrophy, and cell death markers, lowering heart failure risk
C. Studies evaluating renal outcomes							
Semaglutide							
Mann et al^37^	T2D and CKD	RCT, 28 countries An 8-week regimen, Semaglutide 0.25 mg/week for four weeks, increased to 0.5 mg for four weeks, maintained 1.0 mg/week	3534	66.6 years, Male 69.7%	17.4 years	32 kg/m^2^	Trial ended early as it met the primary endpoint. Delayed CKD progression, reduced renal and CV mortality, improved eGFR, fever MACEs, and lower mortality

Abbreviations: CVD: cardiovascular disease; CKD: chronic kidney disease.

A study by Sherr et al^23^ demonstrated that youth with recent-onset T1D exhibit significantly elevated glucagon levels compared to nondiabetic controls during mixed meal tolerance tests, suggesting that suppressing these responses could enhance glycemic outcomes. Brown et al reported a 37% increase in meal-stimulated glucagon levels and a 45% decline in C-peptide function over 12 months in children with newly diagnosed T1D, compared to a 15% increase in fasting glucagon levels, which remained within the normal range. These findings suggest hyperglucagonemia as a potential therapeutic target in T1D.^41^ Fredheim et al^42^ studied a cohort of 129 Danish children (mean age ten years) with T1D over 60 months, finding that postprandial glucagon levels increased 160% from diagnosis and correlated with higher glucose levels and lower C-peptide levels. By addressing both insulin and glucagon dysregulation, GLP-1RAs offer a multifaceted approach to improving glycemic outcomes and overall management of T1D.

Below, we summarize recent studies and meta-analyses evaluating the use of GLP-1RAs in improving glycemic outcomes in adults with T1D.

#### Liraglutide

In the adjunct 1 trial,^25^ adding liraglutide to insulin therapy in 1398 adults with T1D over 52 weeks resulted in significantly reduced HbA1c levels (0.34%-0.54% from an initial 8.2%) and lower insulin doses in the liraglutide 1.8 and 1.2 mg groups.^25^ Mean body weight significantly decreased across all liraglutide groups compared to placebo: 1.8 mg: −4.9 kg, 1.2 mg: −3.6 kg, and 0.6 mg: −2.2 kg.^25^

In the adjunct 2 trial over 26 weeks among 835 individuals with T1D (mean HbA1c 8.1%), adding liraglutide to capped insulin doses significantly reduced HbA1c levels and body weight compared to placebo (liraglutide 1.8 mg: −0.33%; 1.2 mg: −0.22%; 0.6 mg: −0.23%; placebo: 0.01% and mean body weight reductions were −5.1 kg, −4.0 kg, and −2.5 kg for liraglutide doses of 1.8, 1.2, and 0.6 mg, respectively, vs −0.2 kg with placebo).^26^

In a randomized study by Kuhadia et al,^27^ involving 72 individuals (placebo = 18, liraglutide = 54) with T1D, who were overweight or had obesity, the addition of liraglutide (1.2, 1.8 mg) to insulin over a 12-week period resulted in modest reductions in average blood glucose, HbA1c, small reductions in insulin doses, significant weight loss, decreased postprandial glucose concentration, and frequent gastrointestinal (GI) side effects.

In a study by Sherr et al,^43^ adding liraglutide reduced glucose excursions and insulin needs while promoting weight loss, in individuals using closed-loop delivery systems.

#### Exenatide

In a study by Herold et al, 79 participants were randomized to receive exenatide extended release (ER) or placebo for 24 weeks. At week 12, exenatide ER significantly reduced HbA1c levels, particularly in those with detectable C-peptide, and promoted significant weight loss compared to placebo. However, these benefits were not sustained at 24 weeks, indicating limited long-term efficacy. Adverse effects were more common with exenatide ER, but hypoglycemia rates did not increase.^28^

#### Meta-analyses comparing efficacy of glucagon-like peptide-1 receptor agonists (liraglutide, albiglutide, exenatide) in type 1 diabetes

A meta-analysis by Park et al reviewed 24 studies involving 3377 individuals (mean age 39.3 years, 54.8% male, mean diabetes duration 1.8 years, mean A_1c_ 7.9%, mean body mass index (BMI) 26.4 kg/m^2^) to evaluate the efficacy of GLP-1 RAs (liraglutide, albiglutide, exenatide) as adjunctive therapy for T1D. Liraglutide demonstrated significant efficacy in reducing A_1c_ effect sizes (−0.09%/mg), weight (−2.2 kg/mg), and total daily insulin (TDI) (−4.32 IU/mg).^31^ However, higher doses of liraglutide were associated with increased odds of nausea and ketosis. Although newly diagnosed or C-peptide-positive individuals experienced greater A_1c_ reductions with liraglutide, weight loss and insulin reduction benefits were similar.^31^

Another meta-analysis by Tan et al^32^ reviewed 11 randomized controlled trials involving 2856 adults with T1D. The analysis found that GLP-1 RAs (liraglutide, albiglutide, exenatide) led to reductions in HbA1c levels by −0.21% (95% confidence interval [CI] = −0.33 to −0.10), weight by −4.04 kg (−4.8 to −3.27), systolic blood pressure by −2.57 mm Hg (−4.11 to −1.03), and diastolic blood pressure by −1.02 mm Hg (−1.99 to −0.06). In addition, there was a decrease in prandial insulin dose (weighted mean difference of −4.23 IU; 95% CI = −5.26 to −3.20), basal insulin dose (−2.40 IU; −3.93 to −0.87), and total insulin dose (−5.73 IU; −10.61 to −0.86).^32^ Importantly, these benefits were not associated with a risk of severe hypoglycemia, diabetic ketoacidosis (DKA), or severe adverse events but were associated with higher rates of GI adverse events.^32^

#### Semaglutide

A retrospective study by Garg et al^29^ evaluated semaglutide in 50 individuals with overweight or obesity with T1D (92% non-Hispanic white, mean age 42 ± 11 years, duration of diabetes 27 ± 12 years), over a year. In this study, individuals who initiated semaglutide experienced an average weight loss of 15.9 lbs (7.6% of baseline body weight) with a change in BMI of 2.65 kg/m^2^ (a 7.9% reduction from initial BMI), significantly outperforming controls, who gained an average of 2.1 lbs. (1.1% of baseline body weight) at one year. The semaglutide group also showed improved glycemic outcomes including reductions in HbA1c, CGM glucose standard deviation, and coefficient of variation (CV), along with an increase in time in range (TIR).^29^

A letter to the Editor in the *NEJM* by Dandona et al^30^ reported studying ten individuals with T1D (ages 21-39 years, mean A_1c_ 11.7% ± 2.1%, mean fasting C-peptide level 0.65 ± 0.33 ng/mL) who were initiated semaglutide, soon after T1D, alongside dietary changes. Within six months, all individuals were able to eliminate prandial insulin, and seven discontinued basal insulin. After 12 months, their HbA1c levels significantly dropped to 5.7% ± 0.4%, fasting C-peptide levels increased to a mean of 1.05 ± 0.40 ng/mL, TIR according to CGM was 89% ± 3% with a 64% reduction in total insulin dose, minor weight loss, and no episodes of severe hypoglycemia, DKA, or other serious side effects after dose stabilization.^30^

A case report by Raven et al described a 36-year-old female (BMI 29.3 kg/m^2^) with a 27-year history of T1D and undetectable C-peptide, who used semaglutide (0.25 mg weekly increased to 0.5 mg weekly) “off-label.” Over six months, she experienced a 16-kg weight loss and a 36% reduction in insulin dose, with improved HbA1c and reduced glycemic variability, without an increase in hypoglycemia.^3^

Currently, three trials are underway investigating semaglutide in this population (NCT03899402; NCT05819138; NCT05822609).

#### Tirzepatide

A study by Garg et al evaluated the off-label use of tirzepatide (TZT) in 26 adults (mean age 42 ± 8 years, mean BMI of 36.7 ± 5.3 kg/m^2^) over a period of eight months, finding significant reductions in HbA1c (0.59%) and body weight (10.1%) while improving glucose metrics, including TIR. The drug was well tolerated, with only two patients discontinuing, highlighting the need for randomized controlled trials in T1D.^44^

Mendoza et al presented a case report on the use of TZT in a 23-year-old female with T1D and obesity. Over 12 weeks, TZT improved glycemic outcomes with reductions in A_1c_ (from 7.4% to 6.9%), increased TIR (31% to 61%), reduced insulin requirements (81.9 to 57.6 units per day), decreased carbohydrate intake (by 24%), and promoted weight loss (−7 lbs).^45^

There is an ongoing clinical trial (NCT06075667) trial investigating the effect of TZT on adolescent obesity. Providers are also using TZT “off-label” to treat adolescents with T2D and/or obesity.

#### Glucagon-like peptide-1 receptor agonists vs sodium-glucose cotransporter-2 inhibitors

A study by Edwards et al reviewed the use of GLP-1 RAs and sodium-glucose cotransporter-2 inhibitors (SGLT2is) as adjunct therapies to insulin in adults with T1D. After more than 90 days of therapy, GLP-1RAs users showed significant reductions in weight, HbA1c, and TDI over one year.^46^ The SGLT2i users also saw significant reductions in HbA1c and basal insulin but had a higher incidence of DKA.^46^ Both therapies having comparable rates of discontinuation due to adverse effects.^46^

### Cardioprotective Effects of Glucagon-like Peptide-1 Receptor Agonists

#### Semaglutide

In the STEP-1 trial in adults with obesity showed a mean reduction in body weight from baseline to week 68 of −14.9% in the semaglutide group 2.4 mg compared to −2.4% with placebo (Table 2).^34^ The STEP TEENS trial for adolescents with obesity showed significant reductions in BMI of −16.1% at week 68 with semaglutide 2.4 mg compared to 0.6% with placebo.^33^ Both trials demonstrated improvements in cardiometabolic risk factors, including waist circumference, glycated hemoglobin levels, and lipid profiles, compared with placebo.^33,34^

The SELECT trial, a large-scale multicenter randomized trial with 17 604 adults (≥45 years, with obesity, pre-existing cardiovascular disease [CVD], and without diabetes) showed that semaglutide 2.4 mg significantly reduced major adverse cardiovascular events (MACEs) by 20%, including heart attack, stroke, or cardiovascular death, over 40 months of follow-up, compared to placebo.^35^

The ENDIS study examined the effects of empagliflozin and semaglutide on endothelial function and arterial stiffness in 89 participants with T1D and without cardiovascular disease.^4^ Both drugs significantly improved endothelial function, as measured by brachial artery flow-mediated dilation, compared to controls after 12 weeks.^4^ Semaglutide also reduced peripheral resistance, indicating improved arterial stiffness, suggesting potentially superior effects on arterial health.^4^

#### Tirzepatide

A study by Jastreboff et al^36^ assessed the efficacy of once-weekly TZT to placebo in adults with obesity in a phase 3 trial (N= 2539, mean weight of 104.8 kg, BMI of 38.0). At week 72, weight reductions were −15.0%, −19.5%, and −20.9% for TZT doses of 5, 10, and 15 mg, respectively, compared to −3.1% with placebo, with improved cardiometabolic measures.^36^

A study by Taktaz et al assessed the potential cardioprotective effects of TZT through a meta-analysis of major clinical trials. The TZT significantly reduced the risk of MACE.^5^ In cellular experiments using human AC16 cardiac cells exposed to high glucose levels, TZT mitigated the expression of markers associated with cardiac fibrosis, hypertrophy, and cell death.^5^ Bioinformatics analysis further validated these results by revealing TZT’s interactions with pathways involved in apoptosis, fibrosis, and contractility in cardiac cells.

##### Renoprotective effects of glucagon-like peptide-1 receptor agonists

Chronic kidney disease (CKD) is a common complication in individuals with both T2D and T1D (Table 2). In the FLOW trial (NCT03819153),^37,47,48^ a phase 3b study (N = 3534 participants, mean age 66.6 years, with T2D and CKD; average diabetes duration 17.4 years, mean eGFR 47.0 mL/min/1.73 m^2^, 68.2% at high risk for CKD progression), participants were randomized to receive either once-weekly semaglutide 1 mg or placebo alongside standard care.^37^ Novo Nordisk halted the trial early due to positive interim efficacy results, with final outcomes expected in 2024.^37^

## Safety of Glucagon-like Peptide-1 Medications

### Gastrointestinal Issues

Common side effects include nausea, vomiting, and diarrhea, which are typically dose dependent and can be reduced by gradual dose titration. In the STEP-1 trial, 4.5% of participants in the semaglutide group discontinued due to GI events (0.8% in the placebo group).^34^ The STEP TEENS trial showed a higher incidence of GI events with semaglutide than with placebo (62% vs 42%), including cholelithiasis in 4% of participants in the semaglutide group and none in the placebo group.^33^ In the SELECT trial, 16.6% discontinued semaglutide due to GI side effects vs 8.2% in the placebo group, with gallbladder disorders slightly more frequent in the semaglutide group (2.8% vs 2.3%).^35^ Owing to their effect on gastric emptying, individuals with gastroparesis or inflammatory bowel disorders are not good candidates for GLP-1 RAs.

### Hypoglycemia

In the adjunct 1 trial, symptomatic hypoglycemia (episodes classified as severe by ADA criteria or associated with plasma glucose level <56 mg/dL, accompanied by symptoms consistent with hypoglycemia) rates were significantly higher in the liraglutide groups 1.8 and 1.2 mg groups (16.5 events/patient years exposure (PYE) and 16.1 events/PYE, respectively) compared to placebo (12.3 events/PYE).^25^ In the adjunct 2 trial, symptomatic hypoglycemia was higher in the liraglutide 1.2 mg group compared to placebo (21.3 vs 16.6 events/patient/year).^26^

### Hyperglycemia and Ketosis

In the adjunct 1 trial, the proportion of subjects with hyperglycemia with ketosis was 11.2%, 7.5%, and 6.3% for liraglutide 1.8, 1.2, and 0.6 mg, respectively, vs 6.9% for placebo. Liraglutide 1.8 mg showed a statistically significant higher rate compared to placebo (0.28 vs 0.12 events/patient/year).^25^ Eight DKA events were confirmed (three, one, and four cases in liraglutide 1.8, 1.2, and 0.6 mg groups, none in placebo), triggered by factors such as infections, pump malfunctions, or prior DKA history, exclusively occurring only in C-peptide-negative subjects.^25^ In the adjunct 2 trial, hyperglycemia with ketosis (>1.5 mmol/L) was more frequent with liraglutide 1.8 mg compared to placebo (0.5 vs 0.1 events/patient/year), with higher but not statistically significant rates for liraglutide 1.2 and 0.6 mg (0.2 events/patient/year each). These episodes were most common in the first eight weeks.^26^

### Diabetic Retinopathy

Rapid improvements in glycemic outcomes have also been associated with worsening of diabetic retinopathy, as observed in a study involving semaglutide.^49^

### Depression

Observational data suggested a link between GLP-1 RAs and severe depression, but the FDA’s review found no clear relationship.^50,51^ A Swedish Danish study showed no difference in the suicidality between individuals (n = 300 000) starting GLP-1 RAs compared to those beginning SGLT-2 inhibitors, over a follow-up of around 2.5 years, with only 1.5% having a prior diagnosis of anxiety or depression.^52^ In FDA trials of semaglutide (n = 3800), no worsening of depression or suicidality was observed over 68 weeks, although individuals with severe psychiatric histories were excluded.^53^ A World Health Organization’s database linked semaglutide but not liraglutide, to more suicidality reports, a pattern absent after excluding antidepressant users.^54^ These studies suggest that GLP-1 agonists are not tied to suicidality in those without mental health issues, but screening is needed for those at risk.

## Conclusions

The mounting evidence strongly supports incorporating GLP-1 RAs as adjunct therapies to insulin in adolescents with T1D offering benefits such as improved glycemic outcomes, weight reduction, and enhanced cardiovascular and renal health. Given the compelling evidence and the ongoing clinical trials, incorporating GLP-1 RAs as adjuncts to insulin therapy in adults and adolescents with T1D could provide significant clinical benefits. As research in both adolescents and adults continues to progress, the use of GLP-1 RAs should be considered an essential addition to T1D management strategies, offering a promising avenue for improving patient outcomes.
